# Adaptive divergence in shell morphology in an ongoing gastropod radiation from Lake Malawi

**DOI:** 10.1186/s12862-019-1570-5

**Published:** 2020-01-09

**Authors:** Bert Van Bocxlaer, Claudia M. Ortiz-Sepulveda, Pieter R. Gurdebeke, Xavier Vekemans

**Affiliations:** 1 CNRS, Univ. Lille, UMR 8198 – Evo-Eco-Paleo, F-59000 Lille, France; 2 Limnology Unit, Department of Biology, Ghent University, 9000 Ghent, Belgium; 3 Department of Geology, Ghent University, 9000 Ghent, Belgium

**Keywords:** Common garden experiment, Geometric morphometrics, Adaptive radiation, Ampullariidae, Phenotypic plasticity, Differential fitness, Local adaptation

## Abstract

**Background:**

Ecological speciation is a prominent mechanism of diversification but in many evolutionary radiations, particularly in invertebrates, it remains unclear whether supposedly critical ecological traits drove or facilitated diversification. As a result, we lack accurate knowledge on the drivers of diversification for most evolutionary radiations along the tree of life. Freshwater mollusks present an enigmatic example: Putatively adaptive radiations are being described in various families, typically from long-lived lakes, whereas other taxa represent celebrated model systems in the study of ecophenotypic plasticity. Here we examine determinants of shell-shape variation in three nominal species of an ongoing ampullariid radiation in the Malawi Basin (*Lanistes nyassanus*, *L. solidus* and *Lanistes* sp. (*ovum*-like)) with a common garden experiment and semi-landmark morphometrics.

**Results:**

We found significant differences in survival and fecundity among these species in contrasting habitats. Morphological differences observed in the wild persisted in our experiments for *L. nyassanus* versus *L. solidus* and *L.* sp. (*ovum*-like), but differences between *L. solidus* and *L.* sp. (*ovum*-like) disappeared and re-emerged in the *F*_*1*_ and *F*_*2*_ generations, respectively. These results indicate that plasticity occurred, but that it is not solely responsible for the observed differences. Our experiments provide the first unambiguous evidence for genetic divergence in shell morphology in an ongoing freshwater gastropod radiation in association with marked fitness differences among species under controlled habitat conditions.

**Conclusions:**

Our results indicate that differences in shell morphology among *Lanistes* species occupying different habitats have an adaptive value. These results also facilitate an accurate reinterpretation of morphological variation in fossil *Lanistes* radiations, and thus macroevolutionary dynamics. Finally, our work testifies that the shells of freshwater gastropods may retain signatures of adaptation at low taxonomic levels, beyond representing an evolutionary novelty responsible for much of the diversity and disparity in mollusks altogether.

## Background

Evolutionary radiations on oceanic islands and in eco-insular lakes are unique model systems to study population differentiation and speciation. Ecological opportunity, i.e. the availability of varied ecological niches, is widely recognized as a driver of adaptive differentiation and lineage splitting, as exemplified in cichlid fishes [1–3]. Although intuitive in concept, ecological opportunity is difficult to quantify empirically because it depends on the biology of taxa and environmental characteristics [4]. Consequently, testing its contribution to morphological divergence and speciation is complicated, but important to understand underlying mechanisms. Previously, the seeming ubiquity of adaptive radiations urged authors to interpret morphological differences among recently diverged species as indicative of adaptive divergence, regardless of whether hypotheses linking putatively adaptive organismal differences to reproductive barriers and to habitat variation had been tested [3, 5–7]. However, radiations where lineage splitting is caused by genetic drift in small, isolated populations or mutation-order speciation are common as well [8–10], which emphasizes the need to carefully test hypothesized eco-evolutionary mechanisms of morphological differentiation. For many evolutionary radiations across the tree of life accurate knowledge is still lacking on the drivers of diversification and thus how disparity in organismal traits among distant lineages and diversity at a low taxonomic level are related. Ultimately, this knowledge gap precludes deciphering how the fate of species lineages is affected by organismal traits, environmental factors, historical contingency, dispersal and ecological opportunity.

Experimental approaches are a conceptually straightforward way to examine the link between morphological disparity and diversification, but at least for invertebrate radiations they are rarely undertaken. Logistic difficulties to set up such experiments in the wild or to maintain and breed putatively differently adapted species in captivity may explain the overall scarcity of such studies. In gastropods, most experimental work has focused on genetic and environmental determinants of shell shape and color [11–15], on how the presence or absence of predators affects life-history traits [16] and shell shape [17–19], and on the ecological context of invasion success in clonal freshwater snails [20, 21]. Many of these studies report phenotypic plasticity to be commonplace, but a strong taxonomic bias prevails, especially in freshwater gastropods [22]. Direct extrapolation of observations across taxa and study systems has therefore poor prospects, although it is frequently practiced. An example is the polemic on ecophenotypic plasticity versus adaptive punctuated change associated with speciation in the fossil freshwater mollusks of the Turkana Basin [23–28]. Taxa that actually belong to different genera were repeatedly suggested to be ecophenotypic variants in these discussions [29]. Although experimental data on how organismal traits may have influenced diversification are largely lacking, preconceptions on the subject clearly prevail. In the absence of a detailed insight into ecophenotypic plasticity, we cannot accurately discern intraspecific and interspecific variation, which hampers an accurate interpretation of morphological change in both extant and fossil species lineages.

Against this background the ongoing radiation of the dextral, hyperstrophic genus *Lanistes* from the Malawi Basin is a promising study system. It comprises five nominal species that display differentiation at various levels of organization [30–34]. Two of these species, to which we refer here as *L.* sp. (*ovum*-like) and *L.* sp. (*ellipticus*-like), were previously assigned to lineages that occur elsewhere in Africa based on morphological resemblance. However, phylogenetic studies indicated that they are endemic to the Malawi Basin and genetically distinct from these geographically distant lineages [30]. They occur mainly in shallow fringing pools, swamps, satellite lakes and rivers, but also within the lake on shallow sandy to muddy substrates in association with shoreline fringing vegetation [34]. The other three species, *L. solidus*, *L. nyassanus* and *L. nasutus*, are restricted to soft substrates within Lake Malawi, where they presumably evolved [32, 35]. *L. solidus* occurs mainly nearby submerged macrophytes at a depth of ~ 1 to 5 m, whereas *L. nyassanus* occupies open sandy substrates that extend to 35 m in depth, and *L. nasutus* is rare and lives at 40 to 90 m depth [32, 34–37]. Behavioral, anatomical and morphological differences among these species have been related to habitat variation as to 1) wave action and water energy (i.e. shell morphology), 2) the presence of predators (i.e. shell thickness, variation in growth rates, burying behavior, the development of a nocturnal lifestyle), and to 3) substrate-specific food sources (i.e. differences in radulae) [32]. A strong migration barrier has caused substantial geographic differentiation between specimens from the northern and southern regions of the Malawi Basin [31]. Within regions (but not between them) isolation-by-adaptation, i.e. a positive correlation between shell-morphological differences and neutral genetic differentiation, is observed [31]. Environmental analysis of *Lanistes* sampling localities indicates that the major axis of habitat variation relates to habitat stability [31]. The stable habitat occurs within Lake Malawi below the wave base and comprises sandy substrates with limited submerged aquatic macrophytes and detritus. This habitat displays restricted diurnal and seasonal fluctuations in temperature and oxygen concentration. The fluctuating habitat is mainly found in satellite lakes, ponds, inflowing and outflowing rivers. It features important diurnal and seasonal fluctuations in temperature and oxygen concentration, abundant fringing vegetation and detritus. Both habitats also differ in the presence of mollusk predators; see e.g. Fryer [38].

The Malawi Basin contains the only extant *Lanistes* radiation. However, much of its morphological disparity resembles variation found in *Lanistes* fossils from the Chiwondo Beds, i.e. deposits formed in a paleolake that existed ~ 2.5 Ma in the Malawi Basin [30], and in the *Lanistes* radiation of paleolake Obweruka in the Albertine Basin [1]. The observed morphospecies have thus either evolved iteratively, or phenotypic plasticity may have an ancient history in *Lanistes*.

**Fig. 1:**
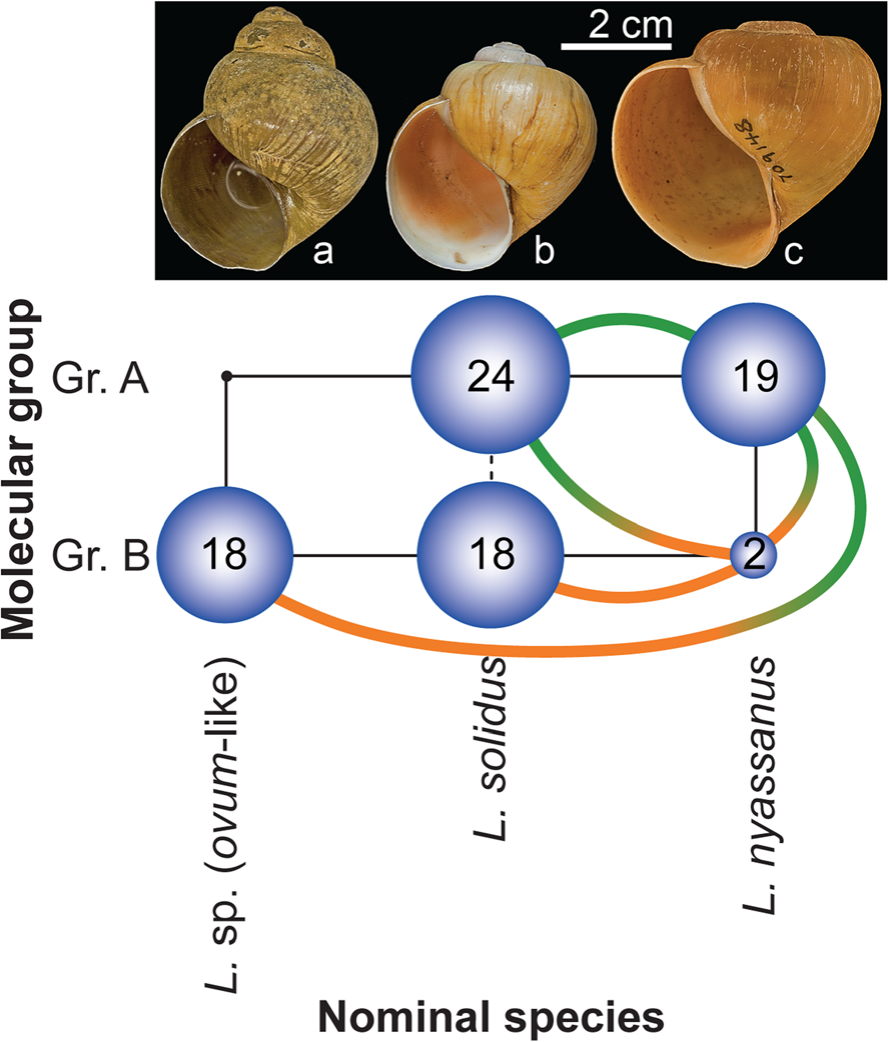
Comparison of nominal species and molecular groups in *Lanistes* from the southern Malawi Basin. A) *Lanistes* sp. (*ovum*-like); B) *L. solidus*; C) *L. nyassanus*. One molecular group (group A) contains exclusively specimens of *L. solidus* and *L. nyassanus*, whereas the other (group B) consists predominantly of *L.* sp. (*ovum*-like) and *L. solidus*. Blue spheres indicate the number of specimens belonging to a certain nominal species and molecular group, whereas colored connections link specimens that come from the same locality but occupy different spheres, i.e. green for group A, orange for group B. Modified from [31]

Here we document the nature of shell-morphological differences in the ongoing *Lanistes* radiation from the Malawi Basin to examine hypotheses on trait utility and supposed differential adaptation. Our experiments focused on the southern region of the Malawi Basin, where three nominal species or morphospecies (*L. nyassanus*, *L. solidus* and *L.* sp. (*ovum*-like)) occur beyond the deep-water species *L. nasutus*, which was not examined here. *Lanistes* sp. (*ovum*-like) occupies fluctuating habitats and *L. nyassanus* stable habitats whereas *L. solidus* occupies habitats of intermediate stability, although they are usually still characterized as ‘stable’. Specimens of these three species belong to two molecular clusters (Fig. 1) [31]. This finding suggests that the previous analysis of a limited set of neutral molecular markers either does not provide sufficient resolution to fully differentiate all nominal species, or that some of these morphospecies may represent ecophenotypic variants. Individuals of the three morphospecies were sampled from six localities where *Lanistes* populations have been genotyped before [31]. Subsequently, we examined the survival of each morphospecies under fluctuating environmental conditions and set up a common garden experiment with conditions that reflected those of the stable, intralacustrine habitat. During this experiment we documented fecundity and shell morphology to examine to what extent morphological differences are genetically determined, and how they relate to fitness. If the observed differences are caused by plasticity, we would expect limited fitness differences among morphospecies throughout the experiment and a shift in the morphospace occupation of *L.* sp. (*ovum*-like) and *L. solidus* towards that of *L. nyassanus*. Alternatively, shell morphology may be predominantly genetically determined, in which case we would anticipate the persistence of morphological differences throughout the experiment. Additionally, an elevated fitness of *L. nyassanus* over at least *L.* sp. (*ovum*-like) would indicate differential adaptation. The experiment was set up in the laboratory because of logistical challenges and to avoid the ecological risk of transplanting closely related species to non-native habitats in the wild.

## Methods

### Material

A total of 184 adult *Lanistes* specimens belonging to three nominal species (*L.* sp. (*ovum*-like), *L. solidus*, and *L. nyassanus*) were collected between 24 and 29 August 2010 from six localities in the southern region of the Malawi Basin, i.e. Lake Malawi and the Shire River (Table 1, Additional file 1: Supplementary text). Specimens were identified to morphospecies following Mandahl-Barth [34] and Berthold [32]. They were labelled with flexible 8 × 4 mm shellfish tags in the field, directly upon sampling. We obtained three, two and one morphospecies at one, three and two localities, respectively. Specimens were kept in morphospecies-specific bags in locality-specific 15 L buckets upon collection and before transfer to the lab. Variation among buckets was minimized by standardizing environmental conditions and treatments. Individuals were fed with JBL Novo Pleco chips and the water was refreshed daily with water directly from the lake. Specimens were individually wrapped in moist toilet paper for transport. After transfer to the laboratory, specimens were placed in pre-installed aquariums, first in individual bags for an acclimation phase of 10 days, and then in their respective lab populations for the experiments.

**Table 1:** Locality information and the number of specimens collected per morphospecies, i.e. *L. o.* = *L.* sp. (*ovum*-like), *L. s.* = *L. solidus* and *L. n.* = *L. nyassanus*

Locality	Latitude (S)	Longitude (E)	*L. o.*	*L. s.*	*L. n.*	Total
Chipalamawamba, Shire River	14.51060	35.26115	9	2	0	11
Palm Beach, Lake Malawi	14.39048	35.21886	0	29	7	36
Maldeco, Lake Malawi	14.33760	35.15712	0	6	0	6
Venice Beach, Lake Malawi	14.09345	34.92739	15	61	0	76
Cape Maclear, Lake Malawi	14.02402	34.84101	6	10	23	39
Chipoka, Lake Malawi	13.98954	34.51854	0	16	0	16
					Total:	184

### Experiments

Experimental populations, i.e. the potentially interbreeding specimens in individual tanks, were constructed per morphospecies by mixing individuals from the various sampling localities to avoid that our experiments would be influenced by variation among sampling localities beyond the focus of our study. Each population was housed in a 63 L aquarium. Practical information on how aquaria were installed and how the experiments were conducted is provided in Additional file 1: Supplementary text. Water conditions were as follows: temperature: ~ 25.9 ± 1.3 °C; conductivity: 1915 ± 376 μS/cm; dissolved oxygen: 5.88 ± 1.30 mg/L; pH: 8.03 ± 0.22. These values reflect natural conditions within Lake Malawi well, except that the conductivity and water hardness were increased in comparison to natural conditions to counteract the dissolution of shells. As mentioned, these lab conditions are very similar to those of the stable natural habitat, i.e. open sand without aquatic vegetation or detritus, and with limited fluctuations in temperature and dissolved oxygen [31].

The experiments were conducted with a total of 132 wild-caught parent individuals as 52 specimens died between capture and the onset of experiments. The survivors were distributed over five *F*_*1*_ experimental populations: two replicate populations for each of *L.* sp. (*ovum*-like) and *L. solidus* and one for *L. nyassanus*, because only 8 wild-caught *L. nyassanus* individuals survived transfer and the adaptation phase. Individuals from *L.* sp. (*ovum*-like) and *L. solidus* were randomly partitioned between the replicates, irrespective of their locality of origin. Experiments were typically started with ~ 10–15 parent individuals per aquarium (but see below). The *F*_*1*_ experiment yielded a total of 242 offspring that reached the subadult stage, allowing more replicates for the *F*_*2*_ phase. In total we established seven *F*_*2*_ experimental populations: two for *L.* sp. (*ovum*-like) and *L. nyassanus*, respectively, and three for *L. solidus*. A total of 138 offspring were obtained in the *F*_*2*_ experiment. One of the two *F*_*2*_ replicates of *L.* sp. (*ovum*-like) did not produce any offspring, despite multiple attempts to restart the experiment.

At the onset of the experiments we had no insight into how many offspring each experimental population would produce, and because crowding could affect growth and morphology, we mitigated its potential effects on our experiments. First, we avoided any effect of crowding on growth and shape by adjusting feeding and maintenance regimes to keep environments similar among tanks and to avoid competition. Furthermore, we created different levels of crowding in the two replicates for *L. solidus* in the *F*_*1*_ experiment (aquaria had 9 and 79 parents, respectively) to qualitatively examine effects. This strategy is suboptimal in that potential differences among replicates may not be attributed to a single factor (i.e. they could relate to crowding, variability in other conditions among replicates, or both), but it is also conservative in that increased variation among replicates, whatever its cause, was incorporated into subsequent statistical tests. Extended experimental procedures required by ampullariid biology are described in Additional file 1: Supplementary text.

### Survival and fecundity

After sampling and before arrival to the lab, individuals experienced conditions that correspond well to those in the fluctuating habitat, i.e. with substantial diurnal changes in temperature and dissolved oxygen. We tracked survival between capture in the wild and the onset of the experiment. As all populations of wild-caught parents reproduced simultaneously, no morphospecies-specific biases exist in our measure of survival. Fecundity was measured as the number of *F*_*1*_ and *F*_*2*_ individuals that survived up to an age of 6 months. As survival and fecundity represent count data, we examined the results with χ^2^ tests.

### Data collecting

Specimens were photographed with a D-SLR camera in apertural view (see Additional file 1: Supplementary text); parents before the experiments, offspring after having been raised for 6 months until they were subadults. The *F*_*2*_ experiment was initiated by picking specimens at random from individual *F*_*1*_ offspring populations before maturity so that pre-experimental copulations were avoided in this generation (see extended experimental procedures in Additional file 1: Supplementary text). These specimens were re-photographed when their population produced eggs, i.e. when they were reproducing adults (which was ~ 9 months after hatching). As already mentioned one of the *F*_*2*_ replicate populations of *L.* sp. (*ovum*-like) did not reproduce, but we re-photographed these specimens at the adult stage too (~ 10 months after hatching). *F*_*2*_ offspring was raised for 6 months and photographed following the same procedure as *F*_*1*_ offspring. Individuals were sacrificed upon termination of each experimental phase and preserved in ethanol for later studies (apart from some wild-caught parents which were used in a hybridization experiment before being sacrificed).

### Geometric morphometrics

Shells were digitized in TpsDig v. 2.31 [39] with 11 landmarks and four open semi-landmark curves. Each of these curves was anchored between two landmarks, and they consisted of 20, 40, 20 and 15 equidistant points respectively, which were obtained via resampling-by-length. Each image contained a scalebar, which we used to convert pixels to cm, so that centroid size unambiguously reflects specimen size. We converted the .tps file with TpsUtil v. 1.75 [40] and imported it into CoordGen8 of the Integrated Morphometrics Package [41]. Variation in scale, orientation and position was removed via Procrustes superimpositioning, after which we defined helper points (11, 21, 11, and 8 per curve, respectively), and slid semi-landmarks along their curves in SemiLand8 via perpendicular projection to the reference of the entire dataset. The resulting dataset with 110 partial Procrustes superimposition coordinates and centroid size was then imported in R v. 3.4.3 [42] for further statistical analyses (with functions of the package stats, unless indicated otherwise).

### Analysis of size

Centroid size was compared among parents and offspring pooled by morphospecies, i.e. a pool of wild-caught and *F*_*1*_ parents and one of *F*_*1*_ and *F*_*2*_ offspring per morphospecies. Minor size differences existed mainly in the parent pool between wild-caught and *F*_*1*_ parents, because *Lanistes* is iteroparous with indeterminate growth. Although growth is much slower in adults the wild-caught specimens cover the whole age spectrum—specimens may live ~ 5–10 years [43]—whereas our *F*_*1*_ parents are all young adults at the onset of reproduction. We checked normality with Shapiro-Wilk tests and the homogeneity of variances with a Bartlett test. Because the assumptions of parametric tests were violated, we used a non-parametric Kruskal-Wallis test and pairwise Wilcoxon rank sum tests with Bonferroni correction to compare size differences among parent and offspring generations of the studied morphospecies.

### Analysis of shell shape

We subjected the geometric morphometric dataset excluding centroid size to non-metric multidimensional scaling (nmMDS) with 1000 random starting configurations, using functions of the packages MASS v. 7.3–48 [44] and vegan v. 2.4–6 [45]. Stress values, i.e. the goodness-of-fit, obtained for nmMDS were compared to the criteria of Kruskal [46] and Clarke [47]: values ≤10 indicate a good fit, those towards 20 or higher indicate gradually increasing chances for misrepresentation and misinterpretation. Subsequently, we examined shape changes directly in the morphospace with functions from geomorph v. 3.0.5 [48, 49].

### Model-based clustering

Patterns of morphospace occupation were examined with model-based clustering using Gaussian finite mixture models as implemented in mclust v. 5.4 [50, 51]. This approach identifies groups based on underlying models of the variance-covariance structure of the data without requiring *a priori* group assignments. Modeling was performed with the expectation-maximization algorithm and model support was evaluated with a Bayesian Information Criterion (BIC). Several new models with complex assumptions on the variance-covariance structure have recently been implemented in mclust, but as some of these models produced clustering schemes that were consistently biologically implausible, we analyzed our dataset with spherical and diagonal models only (see Additional file 1: Supplementary text).

### Statistical comparisons of shape

We tested whether populations grouped by generation (*P*, *F*_*1*_, *F*_*2*_) and by morphospecies differ in morphospace occupation. We first evaluated the assumptions for parametric tests, i.e. multivariate normality and equality of the variance-covariance for each group. Multivariate normality was examined with an *E*-test with 1000 bootstrap replicates using functions of energy v. 1.7–2 [52]. Multivariate homogeneity of group variances was tested with the betadisper function of vegan, and pairwise equality of variance-covariance matrices with our own implementation of Box’s M test. As some assumptions were not met, we used non-parametric permutation tests to examine whether the multivariate means of populations were equal. We carried out a permutational multivariate analysis of variance (perMANOVA) on distance matrices with 10,000 permutations using the adonis function of vegan. Additionally, we also compared the test statistics of 10,000 MANOVA tests on permuted datasets to that of the actual dataset. Both methods gave similar results, so we only report adonis results here. Subsequently, we performed pairwise permutational Hotelling *T*^*2*^ tests with 10,000 permutations in Hotelling v.1.0–4 [53] and Bonferroni correction. These tests trace the significance of differences in the multivariate means of groups, but such differences alone do not necessarily imply that the groups effectively occupy distinct regions of morphospace. Therefore, we examined potential group differences further for morphospecies that were not fully resolved by model-based clustering. Specifically, we used bootstrapping to statistically compare the separation in morphospace between these morphospecies to the variation among biological replicates. We calculated Euclidean distances among morphospecies and replicates and compared these distances with pairwise Bonferroni-corrected Dunn’s tests using functions of dunn.test v. 1.3.5 [54]. We only used 100 bootstraps because we are interested in conservative statistics (the number of bootstraps influences the power of these tests).

### Heritability of shell morphology

We undertook an exploratory analysis of the narrow-sense heritability (*h*^*2*^) of phenotypic traits via parent-offspring regressions. Several caveats are to be considered, however. First, our experiments were designed to allow free mating among the individuals in each experimental population (as required to examine fecundity), and promiscuity hampers establishing parent-offspring relations without invasive genotyping, even for descendants from the same egg cluster. Moreover, our experiments include a contrast between the environment experienced by wild-caught parents before capture and the laboratory conditions in which the *F*_*1*_ and *F*_*2*_ generations were raised. If this cross-generational difference would have generated directional plastic changes rather than changes in the magnitude of phenotypic variation in offspring populations, it may bias our heritability estimates. Estimates from the wild may also be affected by cross-generational environmental heterogeneity, however. On the other hand, our experiments allowed to document offspring morphology at a standardized age so that allometric variation does not confound our results, which is more challenging to control in similar studies from the wild. In summary, the accuracy of our estimates may be limited but because no data exist on the narrow-sense heritability of shell traits in the studied *Lanistes* radiation or even the family Ampullariidae, even coarse estimates could be useful. Given that the father and mother of each offspring individual are unknown, we randomly assigned parents to reconstruct the mid-parent mean. This strategy will underestimate heritability, because parent-offspring associations would deviate increasingly from random as heritability increases. The limitations on parent-offspring assignment imply that our estimates are constructed from patterns across generations rather than contemporaneous parent-offspring associations. After randomly associating parents to offspring we calculated regressions following [55] for each nmMDS axis and each morphospecies with 10,000 bootstraps on mid-parent-offspring assignments. An estimate of the narrow-sense heritability (*h*^*2*^) was then inferred from the obtained summary statistics.

## Results

### Survival and fecundity

**Fig. 2:**
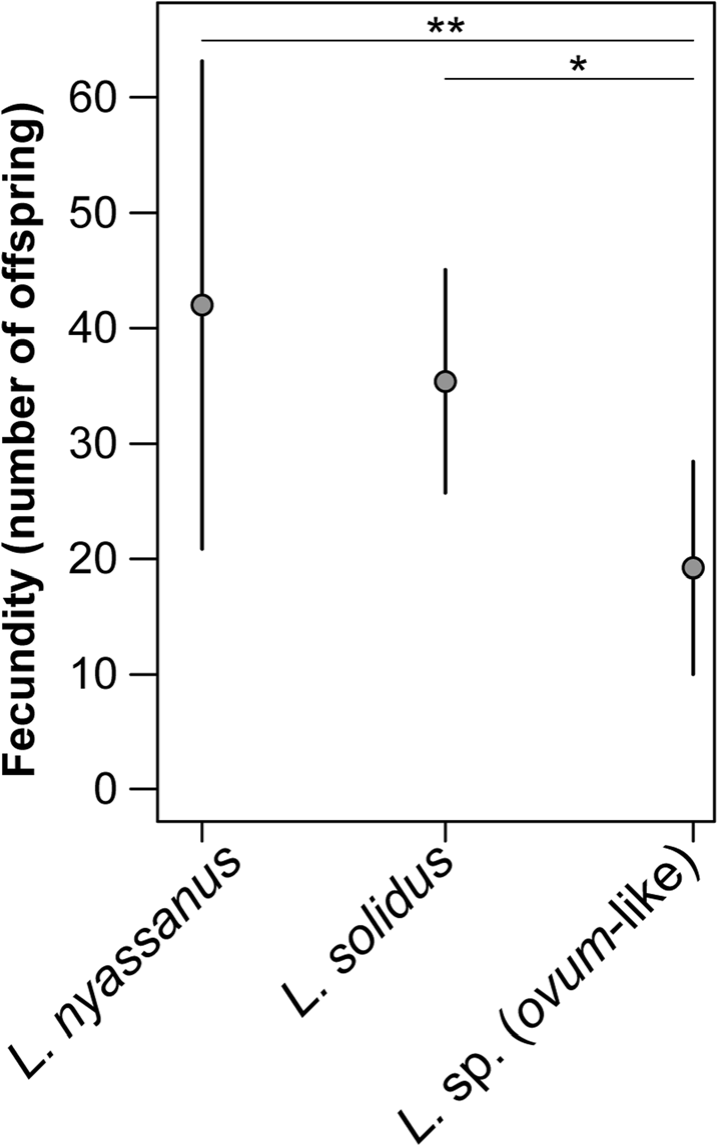
Fecundity of morphospecies during the common garden experiment. Fecundity represents the number of offspring (mean ± standard error) generated by morphospecies averaged over the *F*_*1*_ and *F*_*2*_ generations. Statistically significant differences are indicated with asterisks (0.05 > * > 0.01 > ** > 0.001)

Survival of wild-caught parents differed significantly among morphospecies between sampling and the start of the experiments (27, 71 and 90% for *L. nyassanus*, *L. solidus* and *L.* sp. (*ovum*-like), respectively; Pearson’s χ^2^ test: χ^2^ = 30.062; *df* = 2; *p* < 0.001). In *L. nyassanus* wild-caught specimens displayed dimorphism in body color: some specimens are white-yellow [56], and others dark-brown (as the other morphospecies). None of the yellowish specimens survived after the first 2 weeks in the lab. Mortality during the experiments, i.e. after the acclimation phase, was overall limited, but somewhat higher in juveniles with shell sizes < 1 cm. We found significant differences in fecundity between morphospecies (χ^2^ test for equal probabilities: χ^2^ = 8.504; *df* = 2; *p* = 0.014). *Lanistes* sp. (*ovum*-like) displayed significantly lower fecundity than *L. nyassanus* and *L. solidus* (Fig. 2).

### Centroid size

**Fig. 3:**
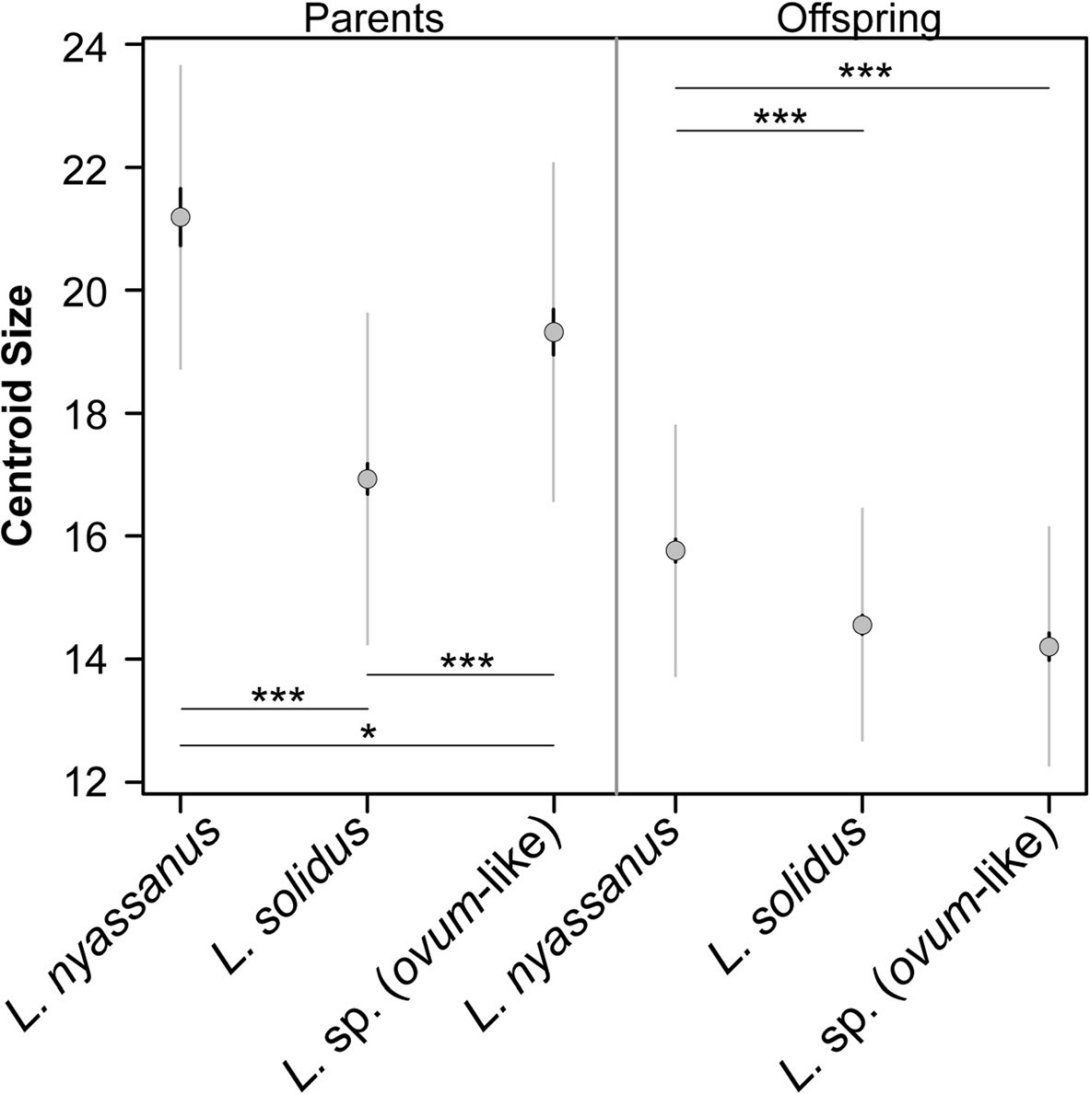
Pairwise comparisons of centroid size by morphospecies for parents (wild-caught and *F*_*1*_) and for offspring (*F*_*1*_ and *F*_*2*_) in our common garden experiment. For parents the centroid size reflects differences in adult body size among morphospecies whereas for offspring it reflects growth rate (all offspring individuals were photographed at an age of 6 months; see material and methods). Black error bars indicate the mean ± standard error, whereas the spread of the data is indicated by the grey bars (mean ± standard deviation). Statistics result from Wilcoxon rank-sum tests with Bonferroni correction. Statistically significant differences are indicated with asterisks (0.05 > * > 0.01 > ** > 0.001 > ***)

Shapiro-Wilk normality tests revealed that shell-size of the parents (pooled wild-caught and *F*_*1*_ parents) and offspring (pooled *F*_*1*_ and *F*_*2*_ offspring) of each morphospecies did not differ substantially from a normal distribution, except for *L. solidus* parents (*W* = 0.963; *p* = 0.003) and *L. nyassanus* offspring (*W* = 0.978; *p* = 0.036). The null hypothesis of homogeneity of the variances was not rejected for parents (Bartlett *K*^*2*^ = 0.453, *df* = 2, *p* = 0.798) or offspring (Bartlett *K*^*2*^ = 0.923, *df* = 2, *p* = 0.630). Significant differences in size existed among parents of all three morphospecies (Kruskal-Wallis rank sum test; χ^2^ = 51.725; *df* = 2; *p* < 0.001). Pairwise comparisons indicated significant size differences among all morphospecies, but the main difference was that *L. solidus* is smaller than *L.* sp. (*ovum*-like) and *L. nyassanus* (Fig. 3). Significant differences in growth rate are observed among the offspring of all three morphospecies (Kruskal-Wallis rank sum test; χ^2^ = 31.391; *df* = 2; *p* < 0.001), but pairwise comparisons indicate that significant differences only exist between *L. nyassanus* versus *L. solidus* and *L.* sp. (*ovum*-like) (Fig. 3).

### Geometric morphometrics

The stress value obtained for nmMDS in two dimensions was substantial (14.71), and, as such, we compared the 2D morphospace occupation with that in 3D which generated less stress (9.26). Overall, we found that the displacement of specimens along the first two axes is limited between both nmMDS analyses (Additional file 1: Figure S1), and exploration of the third axis did not reveal any additional patterns of group separation. As such, nmMDS in 2D is robust for our dataset, which was further confirmed by comparison to principal component analysis (not shown).

**Fig. 4:**
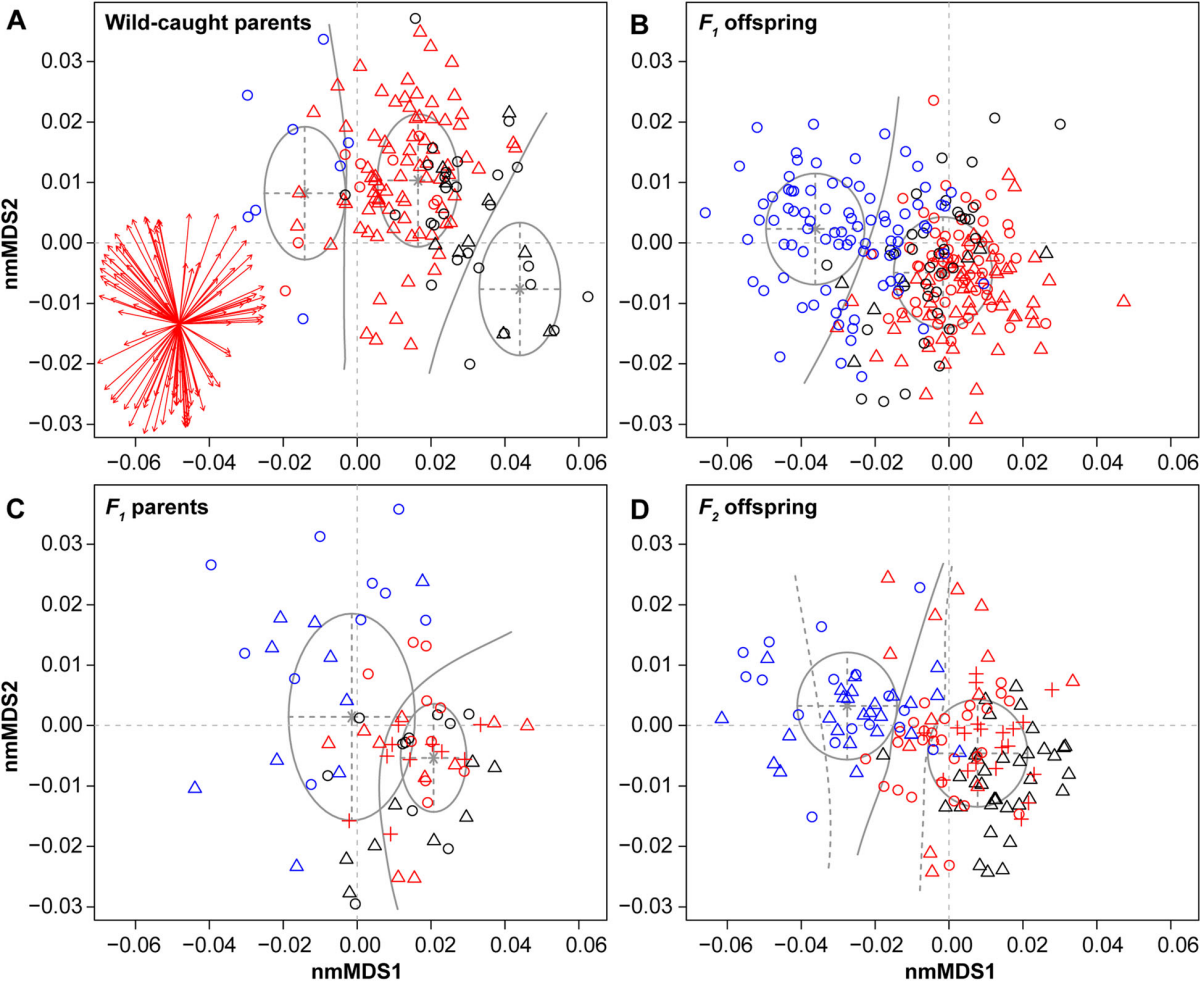
Morphospace occupation for all specimens in our common garden experiment. **a** wild-caught parents; **b** *F*_*1*_ offspring; **c** *F*_*1*_ parents; **d** *F*_*2*_ offspring. Colors indicate morphospecies (blue = *L. nyassanus*, red = *L. solidus*, black = *L.* sp. (*ovum*-like)), whereas symbols (circles, triangles, crosses) indicate replicates. The biplot is represented at 75% of its actual size and indicates the contribution of morphometric components to the morphospace. Grey spheres and solid separation lines indicate the best-supported solutions of model-based clustering with Gaussian mixture models (see Fig. 6); for *F*_*2*_ offspring (**d**) the three-group model is added with dashed lines

**Fig. 5:**
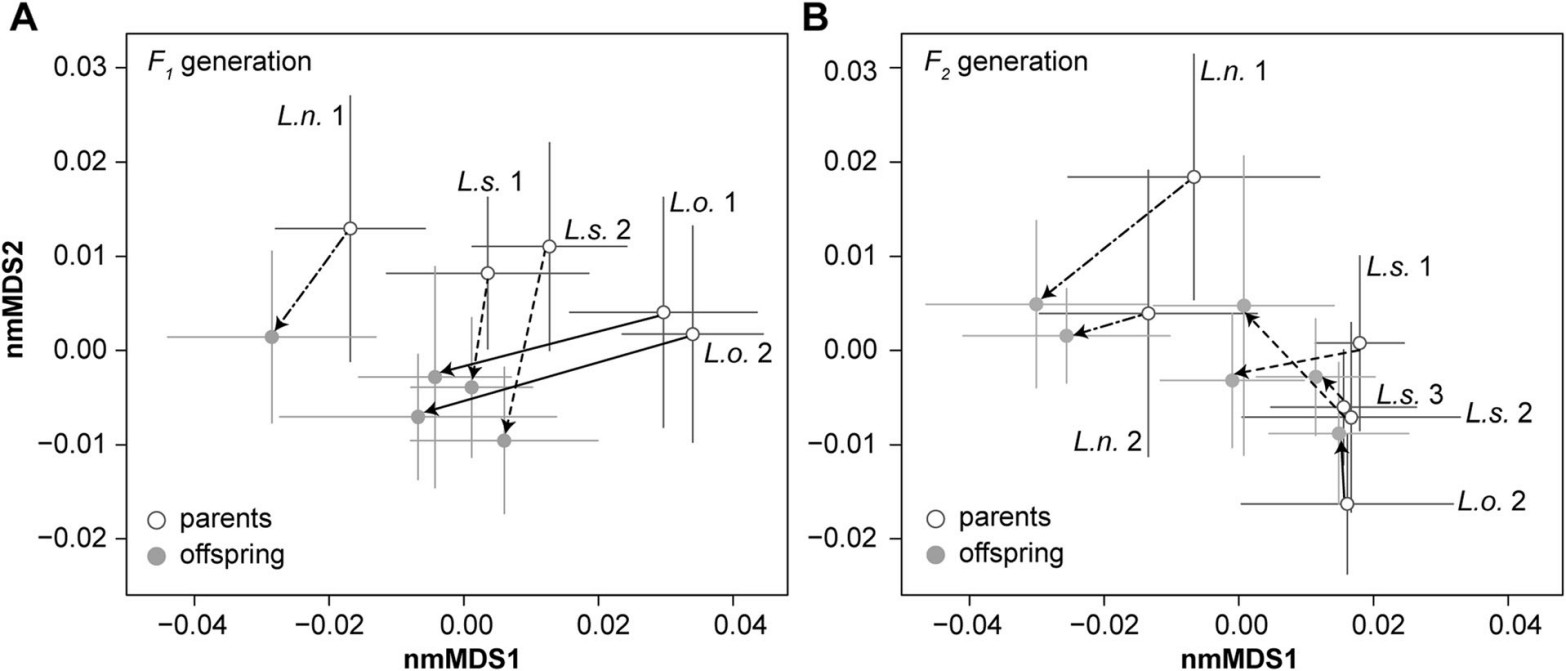
Morphospace changes in replicates for the *F*_*1*_ and *F*_*2*_ generations. Morphospace changes (indicated with arrows) are reconstructed from the morphospace occupation of populations (mean ± one standard deviation). All arrows for replicates point in a similar direction and are quasi-parallel, except for the slightly different trajectory of *L. solidus* rep. 1 in comparison to the other replicates in the *F*_*2*_ generation. Replicates thus show overall very similar changes, indicating that the design of our experiment was robust

The morphospace occupation of all three morphospecies throughout the common garden experiment is illustrated in Fig. 4. Morphological changes along nmMDS1 reflect shell translation parameters and thus the height of the spire, whereas nmMDS2 captures other apertural changes (Additional file 1: Figure S2). Substantial differences in morphospace occupation of the three morphospecies existed in the wild-caught parents, although some overlap is observed, especially between *L. solidus* and *L.* sp. (*ovum*-like) (Fig. 4a). As mentioned, these latter two morphospecies differ significantly in size (Fig. 3). Comparing the morphospace occupation of parents and offspring indicated highly consistent changes among replicates for all morphospecies (Fig. 5). Consistent patterns among replicates indicate that crowding did not affect growth or shape under our precautions of *ad libitum* feeding and adjusted maintenance.

There was a general tendency of the 6-month-old lab-offspring to plot along lower values on nmMDS2 than their parents (Fig. 4b, d). Upon comparing morphospecies, we observed substantial differences between *L. nyassanus* versus *L. solidus* and *L.* sp. (*ovum*-like) in both *F*_*1*_ and *F*_*2*_ descendants. The differences between *L. solidus* and *L.* sp. (*ovum*-like) strongly diminished in the *F*_*1*_ offspring to re-increase in the *F*_*2*_ offspring (Fig. 4b-d). Indeed, the offspring of *Lanistes* sp. (*ovum*-like) overlapped completely with that of *L. solidus* in the *F*_*1*_ generation, but not in the *F*_*2*_ generation. This result mainly relates to changes in the morphospace occupation of *L.* sp. (*ovum*-like) over generations. The *F*_*2*_ offspring of *L.* sp. (*ovum*-like) clustered along more positive values on nmMDS 1 than that of *L. solidus*, as was the case in wild-caught parents, whereas its *F*_*1*_ offspring was positioned between those of *L. solidus* and *L. nyassanus* on nmMDS 1 (Fig. 4).

### Model-based clustering

**Fig. 6:**
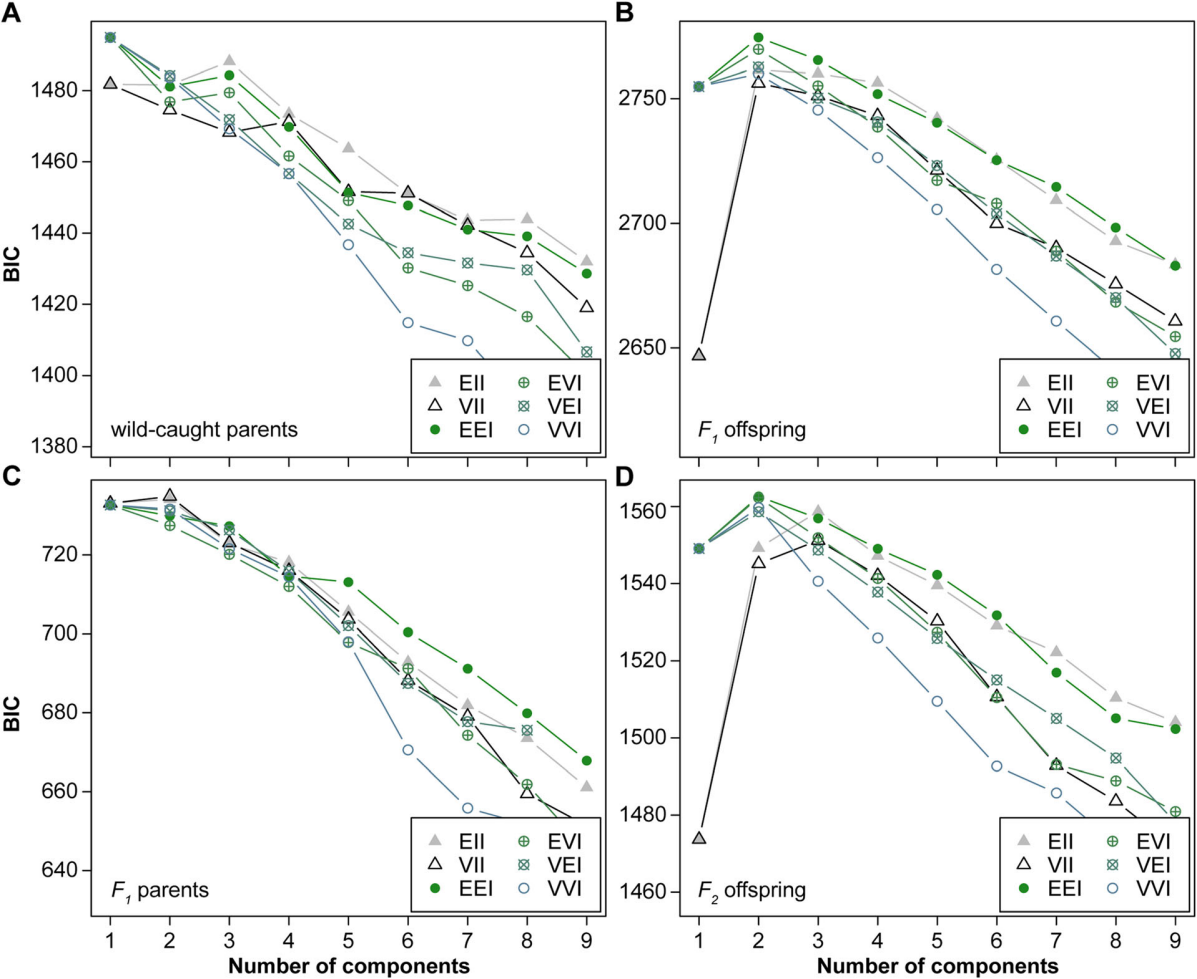
Model support for the various normal mixture models in function of the number of clusters (1–9) considered. A Bayesian information criterion (BIC) was used to examine the fit of clustering solutions proposed by 6 spherical and diagonal normal mixture models to the morphospace occupation for all groups in the experiment: **a** wild-caught parents; **b** *F*_*1*_ offspring; **c** *F*_*1*_ parents; **d** *F*_*2*_ offspring. Scenarios with 1 to 9 clusters were considered; models are explained in Additional file 1: Supplementary text

The best-supported solutions of model-based clustering are indicated in Fig. 4, with a comparison of model support in Fig. 6. For the wild-caught parents a one-group model was preferred (Fig. 6a), which relates to the strongly different representation of the various morphospecies at the start of the experiments, partly because of marked differences in survival (see above). However, three models (EII, EEI and EVI) peaked for a solution with three clusters (ΔBIC = 6.73 in comparison to the one-group model), with better support for the three-group than the one-group solution in one case (EII). This three-group model differentiates the three morphospecies well, despite marked differences in their representation in the dataset. For the *F*_*1*_ offspring generation, a two-group model presented a stable outcome for all considered clustering methods (ΔBIC = 9.06 over models with three groups; Fig. 6b). One of these groups coincides with *L. nyassanus*, the other with *L. solidus* and *L.* sp. (*ovum*-like). The classification for the *F*_*1*_ parents was very similar to that of the *F*_*1*_ offspring. Two-group models again received most support (Fig. 6c), and again separated *L. nyassanus* from *L. solidus* and *L.* sp. (*ovum*-like). However, as the number of specimens included per group was more limited, the support for the two-group model over a one-group model was smaller for the *F*_*1*_ parents in comparison to the *F*_*1*_ offspring (ΔBIC = 1.75). For some algorithms (all but EII and VII) a one-group model received marginally better support than a two-group model. For the *F*_*2*_ offspring, a two-group model was the best-supported outcome and models with a single group are highly unlikely (Fig. 6d). However, two out of the six tested models (EII and VII) gave stronger support for three groups than for two groups. The difference between the best two and three group models is ΔBIC = 3.92. The two-group model is illustrated in Fig. 4 with solid lines, whereas the separations between the groups of the best-supported three-group model are indicated with dashed lines. The two-group solution again reconstructed the separation between *L. nyassanus* versus *L. solidus* and *L.* sp. (*ovum*-like). In contrast, the three-group model subdivided *L. nyassanus* rather than separating *L. solidus* from *L.* sp. (*ovum*-like).

### Statistical analysis

Whereas shape differences between *L. nyassanus* versus *L. solidus* and *L.* sp. (*ovum*-like) were consistently recovered via model-based clustering, the differences between *L. solidus* and *L.* sp. (*ovum*-like) were subtler and therefore we tested them with multivariate statistics. Multivariate normality was not rejected for any of the morphospecies × generation groups, except for the *F*_*2*_ offspring of *L. nyassanus* (energy = 0.967, *p* = 0.044; Additional file 1: Table S1). Homogeneity of the variance-covariance of these groups was rejected, however (*F* = 2.845, *df* = 11, *p* = 0.001), and bonferroni-corrected pairwise Box’s *M* tests indicated that 27 out of 66 pairs showed significant differences in variance-covariance (for 21 pairs: 0.05 > *p* > 0.001; for 6 pairs: *p* < 0.001). Permutational MANOVA indicated that significant differences exist in the means of morphospecies in the morphospace (*F* = 12.641, *df* = 1, *p* < 0.001). The results of Bonferroni-corrected pairwise permutation tests are illustrated in Table 2. All three morphospecies differed significantly in the wild-caught parents as well as in the *F*_*1*_ and *F*_*2*_ offspring generations, but *L. solidus* and *L.* sp. (*ovum*-like) could not be distinguished in the *F*_*1*_ parent generation. Other pairwise comparisons across generations show highly significant differences, except between multiple generations of *L. nyassanus*, indicating that this morphospecies overall had a stable position in morphospace. The morphospace occupation of *F*_*2*_ offspring of *L. solidus* did not differ significantly from that of *F*_*1*_ offspring of *L. solidus* (and *L.* sp. (*ovum*-like)), suggesting that it also occupies a relatively stable position in morphospace. The *F*_*2*_ offspring of *L.* sp. (*ovum*-like) did not differ significantly in shape from the *F*_*1*_ parents of *L. solidus* and *L.* sp. (*ovum*-like). This latter finding suggests that after strongly different shape changes in *L.* sp. (*ovum*-like) during the first and second generations (almost exclusively along nmMDS1 and nmMDS2, respectively) the occupation of this species in morphospace is stabilizing.

**Table 2:** Permuted *T*^*2*^ statistic (lower triangle) and the associated Bonferroni-corrected *p*-values (upper triangle) for morphospecies × generation comparisons (*n* = 66). Significant *p*-values are indicated in boldface. WP = wild-caught parent, F1O = *F*_*1*_ offspring; F1P = *F*_*1*_ parent; F2O = *F*_*2*_ offspring, Lnya = *L. nyassanus*, Lsol = *L. solidus*, Lov = *L.* sp. (*ovum*-like)

	WP_Lnya	WP_Lsol	WP_Lov	F1O_Lnya	F1O_Lsol	F1O_Lov	F1P_Lnya	F1P_Lsol	F1P_Lov	F2O_Lnya	F2O_Lsol	F2O_Lov
WP_Lnya		**< 0.001**	**< 0.001**	**0.040**	**< 0.001**	**< 0.001**	1.000	**< 0.001**	**< 0.001**	0.290	**< 0.001**	**< 0.001**
WP_Lsol	45.85		**< 0.001**	**< 0.001**	**< 0.001**	**< 0.001**	**< 0.001**	**< 0.001**	**< 0.001**	**< 0.001**	**< 0.001**	**< 0.001**
WP_Lov	92.99	73.69		**< 0.001**	**< 0.001**	**< 0.001**	**< 0.001**	**< 0.001**	**< 0.001**	**< 0.001**	**< 0.001**	**< 0.001**
F1O_Lnya	15.82	387.35	439.87		**< 0.001**	**< 0.001**	**< 0.001**	**< 0.001**	**< 0.001**	1.000	**< 0.001**	**< 0.001**
F1O_Lsol	66.67	180.53	193.65	315.26		**< 0.001**	**< 0.001**	**< 0.001**	**< 0.001**	**< 0.001**	0.251	**< 0.001**
F1O_Lov	39.60	73.26	148.66	88.50	22.10		**< 0.001**	**< 0.001**	**< 0.001**	**< 0.001**	0.482	**< 0.001**
F1P_Lnya	1.49	49.23	103.67	33.78	92.65	33.90		**< 0.001**	**< 0.001**	**< 0.001**	**< 0.001**	**< 0.001**
F1P_Lsol	82.49	58.20	37.66	219.16	32.04	66.26	86.72		1.000	**< 0.001**	**< 0.001**	1.000
F1P_Lov	85.38	73.48	47.01	152.29	21.13	66.50	83.73	5.74		**< 0.001**	**0.0066**	1.000
F2O_Lnya	12.94	248.16	336.71	1.07	227.70	82.96	20.49	186.81	154.01		**< 0.001**	**< 0.001**
F2O_Lsol	36.56	60.92	129.25	183.37	12.15	10.75	41.57	27.46	27.51	134.34		**< 0.001**
F2O_Lov	117.58	109.59	77.22	259.14	31.11	92.78	125.10	5.30	0.71	242.55	38.52	

A greater morphospace distance was observed between *L. solidus* and *L.* sp. (*ovum*-like) in the *F*_*2*_ offspring (13.75 × 10^− 3^ ± 2.38 × 10^− 3^ nmMDS units) than in the *F*_*1*_ offspring (8.58 × 10^− 3^ ± 1.52 × 10^− 3^ nmMDS units; *Z* = − 11.082, *p* < 0.001). This finding is also corroborated by the pairwise permutation tests (Table 2) which indicated that *F*_*1*_ parents displayed no significant differences in morphospace, but their *F*_*2*_ offspring did. In the *F*_*1*_ offspring the morphospace distance between *L. solidus* and *L.* sp. (*ovum*-like) was not significantly different from that between replicates within morphospecies (7.96 × 10^− 3^ ± 2.63 × 10^− 3^ nmMDS units; *Z* = − 1.288, *p* = 0.593). In the *F*_*2*_ generation, however, the morphospace distance between *L. solidus* and *L.* sp. (*ovum*-like) was significantly larger than that between replicates (11.77 × 10^− 3^ ± 2.70 × 10^− 3^ nmMDS units; *Z* = − 3.922; *p* < 0.001).

### Heritability of shell morphology

**Fig. 7:**
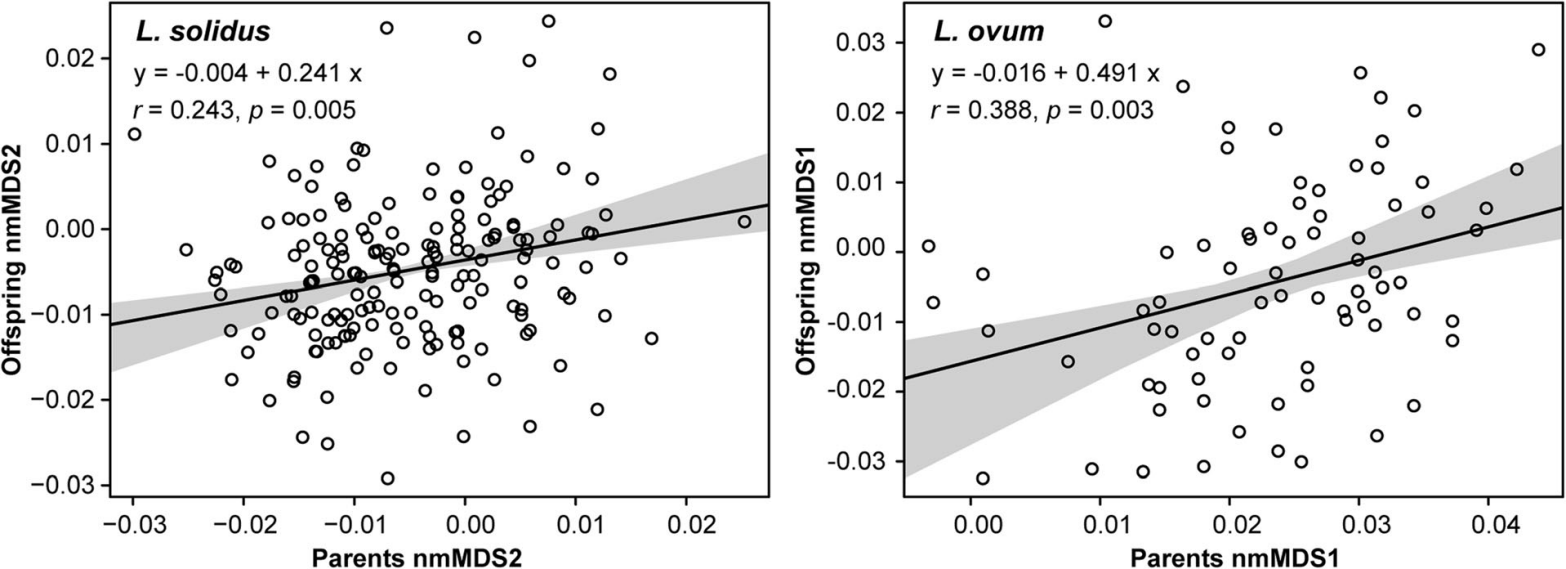
Heritability of shell morphology as inferred from regressions of mid-parents versus offspring. The axes of nmMDS were used as proxy for shell morphology, each of which represents a module of which shape variation is illustrated in Additional file 1: Figure S2. Individual points represent randomly constructed associations of mid-parents and offspring for a single morphospecies in a single bootstrap replicate. Each point represents thus either an association of wild-caught parents and *F*_*1*_ offspring or *F*_*1*_ parents and *F*_*2*_ offspring. The regression represents the summary statistics from 10,000 bootstraps on parent-offspring associations, with the mean in black and the 95% confidence interval in grey

For *L. nyassanus* nmMDS1 and 2, *L. solidus* nmMDS1 and *L.* sp. (*ovum*-like) nmMDS2 no estimate on heritability could be obtained from randomized mid-parent-offspring regressions, i.e. randomizations obliterated potentially existing patterns. However, for *L. solidus* nmMDS2 and *L.* sp. (*ovum*-like) nmMDS1 bootstrapped parent-offspring regressions retained significant trends regardless of our randomizations (Fig. 7), with an average heritability of *h*^*2*^ = 0.24 (95% CI: 0.15–0.43) and *h*^*2*^ = 0.49 (95% CI: 0.32–0.94), respectively. These values and their 95% CI cover much of the heritability range previously reported for a variety of morphological traits in other taxa [57, 58].

## Discussion

### Substantial differences in survival and fecundity

Morphospecies from progressively more stable natural habitats displayed significantly increased mortality under the fluctuating conditions that were imposed during transfer between sampling and the start of the experiment. Indeed, substantial diurnal fluctuations in oxygen and temperature occurred in transportation buckets compared to intralacustrine habitats at several meters of depth, but these conditions resemble diurnal variation in fluctuating natural habitats well. In *L. nyassanus* differences in survival also seem to be correlated with body color, suggesting that one color morph is more resistant than the other, or that various populations of *L. nyassanus* are adjusted to finer habitat differences than examined thus far.

The conditions in our common garden experiment reflected, as previously explained, a high level of environmental stability in comparison to the total range of freshwater habitats occupied by *Lanistes* in the Malawi Basin [31]. During the experiments we found that fecundity was highest in morphospecies from stable natural environments, i.e. *L. nyassanus* and *L. solidus*, and significantly lower in *L.* sp. (*ovum*-like), which occupies strongly fluctuating natural habitats. The differences in survival and fecundity indicate marked variation in the tolerance among morphospecies to a range of environmental stressors, and thus fitness. The lower survival rate of *L. nyassanus* during transport is consistent with its stenotopic natural habitat. However, the lower fecundity of *L.* sp. (*ovum*-like) suggests that this morphospecies is not an opportunistic generalist, as previously presumed [33, 34], but rather locally adapted to the fluctuating habitat. Our experiments were not set up as a formal test of local adaptation, which would require fully reciprocal tests of survival and fecundity under various environmental conditions [59]. Nevertheless, our results on survival and fecundity follow the pattern expected for local adaptation, as did the previously recovered pattern of isolation by adaptation [31] and some of our current morphometric results (see below).

### Size differences between morphospecies

Our *F*_*1*_ and *F*_*2*_ offspring specimens were imaged after 6 months, and over this period *L. nyassanus* reached a significantly larger size than *L. solidus* and *L.* sp. (*ovum*-like). Two hypotheses could explain why *L. nyassanus* grew faster than the other two morphospecies in our experiments. First, differences in growth rate may reflect differences among morphospecies in a plastic response to the availability of resources. Alternatively, *L. nyassanus* may have previously experienced selection for faster growth to shorten the period during which juveniles are vulnerable to predators. Whereas we cannot exclude plasticity entirely, it is unlikely the sole contributing factor because growth likely reflects food availability more than abiotic conditions, and food was provided *ad libitum* to all morphospecies. Additionally, increased growth rates in juvenile *L. nyassanus* have been independently documented in the wild [36]. Furthermore, the hypothesis of intense predation in intralacustrine environments is consistent with the occurrence of crabs [60] and molluscivore cichlids [38], the cryptic behavior of hiding within vegetation during the day observed in juveniles of *L. solidus* [36], and the nocturnal lifestyle of *L. nyassanus* [32, 43]. This lifestyle moreover has a strong genetic basis in *L. nyassanus* (Van Bocxlaer & Ortiz-Sepulveda, pers. obs.). Morphological data are also consistent with the predatory-avoidance hypothesis: *L. solidus* and to lesser extent *L. nyassanus* have thick-walled shells that are somewhat and strongly inflated, respectively [32–34]. Thick-walled rotund shells are harder to crush by shell-crushing predators than thin-walled high-spired shells, such as those of *L.* sp. (*ovum*-like) [18, 60].

### Geometric morphometrics

The similar changes in morphospace occupation from parents to offspring among replicate populations indicate that our common garden experiment was designed robustly, and that different levels of crowding did not affect shell shape. This context allows the interpretation of shape changes between parents and offspring. We observed a general tendency of the 6-month-old lab-offspring to plot along lower values on nmMDS2, which we relate to size differences and the more standardized age cohort in *F*_*1*_ and *F*_*2*_ offspring in comparison to the wild-caught parents.

The strongest separation in morphospace occurred between *L. nyassanus* versus *L. solidus* and *L.* sp. (*ovum*-like), which persisted in all parent and offspring generations, as supported by model-based clustering. Overall, model-based clustering provides a good insight into how data is structured in the morphospace, but it is sensitive to the amount of data available and how specimens are distributed over groups. As such, the single-group outcome of various models for the wild-caught parents is to be interpreted with caution because morphospecies were differently represented in this generation, partly owing to differences in survival. Morphological differences between *L. solidus* and *L.* sp. (*ovum*-like) are subtler and not picked up by model-based clustering. This result could either imply that no important biological differences exist, or that they do, but that the morphospecies plot so close to one another in morphospace that the available data are insufficient to reconstruct the separation. Bootstrapping and statistical comparisons indicated no statistically significant differences between both morphospecies in the *F*_*1*_ generation, but in the *F*_*2*_ generation they are separated by a distance that is significantly larger than that among replicates. A complication of this test is that one of the *F*_*2*_ replicates for *L.* sp. (*ovum*-like) did not produce offspring, and thus that the distance among replicates is established only from replicates of *L. solidus*. However, the absence of reproduction in one replicate for *L.* sp. (*ovum*-like) seems to reflect stressful experimental conditions for this species rather than an artifact. Our morphometric results suggest that the similarities between *L. solidus* and *L.* sp. (*ovum*-like) in the *F*_*1*_ offspring may be caused by plastic trans-generational effects, and that re-differentiation in the *F*_*2*_ generation is an expression of genetic differences as these effects wane. Our heritability estimates tentatively confirm this result, although they should be treated with caution due to the methodological constraints indicated above.

Some aspects of the observed morphospace patterns corroborate expectations for local adaptation. *Lanistes nyassanus*, the morphospecies inhabiting stable natural habitats occupied a very stable morphospace position during our experiments, suggesting that environmental changes from the wild towards the lab were minor for this species. In contrast, the *WP*-*F*_*1*_ offspring morphospace displacements for *L.* sp. (*ovum*-like) were substantially larger than those of *L. nyassanus* and *L. solidus*, corroborating the dissimilarity of laboratory and natural conditions for this species. Multiple mechanisms may contribute to these large displacements, including parental effects such as trans-generational plasticity caused by predation in the wild-caught parental population (see e.g. [61, 62]), or a potential selection pressure, which may also have caused decreased fecundity in comparison to the other morphospecies.

Finally, we observed increased morphological variation in the *F*_*1*_ parents of *L. nyassanus* in comparison to the wild-caught parents, which was not observed for other morphospecies. This change in variability may hint to differences in the selective forces operating in the wild versus the lab. Although the experiments were designed to reflect the abiotic conditions of stable environments in Lake Malawi, they excluded predation. Increased morphological variability in *F*_*1*_ parents of *L. nyassanus* may relate to a release from predation, which may selectively remove morphological variation in the wild.

### Evolutionary significance of shell traits

The shell and radula are key innovations responsible for much of the macroevolutionary success of mollusks [63, 64]. At a low taxonomic level, however, phenotypic plasticity in shell traits is pervasive [22], indicating the need to document the extent to which morphological variation is determined by genetic and environmental factors. Here we show that although some of the shell-morphological variation in the ongoing *Lanistes* radiation of the Malawi Basin may have an ecophenotypic nature, most differences are genetically determined. Whether shell morphology has induced trait-dependent diversification, much like the pharyngeal jaws in cichlids [1], requires further study. These jaws represent a key innovation of cichlids [65, 66], but they also display remarkable levels of phenotypic plasticity, which may have played an important role in adaptive radiation [67, 68]. Some authors [69] have argued that the occurrence of radiations with replicated differentiation in traits prone to environmental variation highlights a role for plasticity in diversification, but the issue is debated [70]. Indeed, iterative radiations may rather reflect recurrent patterns of selection in time and space and/or a role for constraints, developmental or otherwise, in restricting the number of repertoires of evolvability. In any case, iterative and parallel evolution of morphotypes have been observed in various freshwater mollusk families and cichlids alike throughout the East African Rift [1, 30]. Our data on the ongoing *Lanistes* radiation from the Malawi Basin strongly suggests that the morphological variation observed in fossil assemblages of *Lanistes* in the Malawi and Albertine Basins represents multiple species. The fact that *L.* sp. (*ovum*-like) was able to cope with the stable habitat conditions in our experiments may hint to a role for plasticity in the early stages of differentiation, but more work is required to address whether the morphospecies is able to cope with all aspects of the natural stable habitat, including predation.

### Species-level differentiation and potential for assortative mating

The frequent sympatry of *Lanistes* morphospecies in the Malawi Basin (e.g. Table 1) and the limited extent to which morphological differences can be explained by phenotypic plasticity as documented in our study suggest that gene flow between the morphospecies is restricted, as it would counteract the here observed morphological differentiation. The previously documented pattern of isolation-by-adaptation also contradicts pervasive gene flow [31], and as such we may wonder to what extent hybridization occurs in the wild. Beyond isolation-by-adaptation, and potentially intrinsic barriers such as Dobzhansky-Muller incompatibilities, assortative mating caused by the observed differences in activity patterns (diurnal in *L.* sp. (*ovum*-like), nocturnal in *L. nyassanus*, and seemingly intermediate in *L. solidus*) may cause prezygotic isolation. Comprehensive analyses based on genome-wide data (e.g. SNPs) would be invaluable to infer demographic scenarios of divergence [71–73], speciation mechanisms, and how widespread highly differentiated regions occur along the genome. The AFLP loci that have currently been examined (*n* = 201) unlikely cover the genome of *Lanistes* sufficiently well to address this question [74]. This issue may also lie at the basis of the incongruence between two molecular groups and the three recognized morphospecies, in particular for *L. solidus* for which individuals were assigned to both molecular groups (Fig. 1). If most of our markers do not cover the regions that drive genomic divergence, we cannot expect to be able to fully differentiate these species. Hence, genomic studies are particularly promising to further examine this radiation.

## Supplementary information

**Additional file 1:** This additional file contains supplementary information to the main text and consists of supplementary text, table S1, figure S1 and S2. **Supplementary text** includes additional methodological descriptions and results. The additional methodological descriptions contain a description of sampling procedures, technical information on how aquaria were set up, extended experimental procedures that were required by ampullariid biology, information for reproducibility of morphometric data collecting, and descriptions of the models that were used in model-based clustering. Additional results consist of direct observations on the reproduction of *Lanistes*. **Table S1.** Results of *E*-tests of multivariate normality per morphospecies and generation. **Figure S1.** Comparison of the morphospace occupation reconstructed by non-metric multidimensional scaling (nmMDS) in 2 and 3 dimensions along nmMDS1 and 2, with vectors representing how individual specimens are displaced from the 3D configuration to the 2D configuration. **Figure S2.** Geometric morphometric shape changes along nmMDS1 and 2.

**Additional file 2:** Supplementary dataset on survival, indicating the number of specimens per morphospecies that survived or died between capture in the wild and the onset of the experiments.

**Additional file 3:** Supplementary dataset indicating general fecundity in our experiments with the number of parents and offspring specimens in each experimental population (in rows). Group assignments furthermore indicate the nominal species to which each experimental population belongs.

**Additional file 4:** Supplementary dataset indicating geometric morphometric data, i.e. 110 partial Procrustes superimposition coordinates and centroid size, for all specimens analyzed in this paper. Each row contains a specimen, with identifications to nominal species, the generation to which the specimen belongs, the initial aquarium number as well as various group identifiers.

## Data Availability

The datasets for all analyses, i.e. data on survival, fecundity and geometric morphometrics including centroid size are provided as Additional files 2, 3 and 4.
