# Supplementary material for: Adaptive divergence in shell morphology in an ongoing gastropod radiation from Lake Malawi
Source: BMC Evol Biol. 2020 Jan 9;20:5. doi: 10.1186/s12862-019-1570-5 (PMC6953155; doi:10.1186/s12862-019-1570-5)
Supplement: Supplementary file 1 — Additional file 1: This additional file contains supplementary information to the main text and consists of supplementary text, table S1, figure S1 and S2. Supplementary text includes additional methodological descriptions and results. The additional methodological descriptions contain a description of sampling procedures, technical information on how aquaria were set up, extended experimental procedures that were required by ampullariid biology, information for reproducibility of morphometric data collecting, and descriptions of the models that were used in model-based clustering. Additional results consist of direct observations on the reproduction of Lanistes. Table S1. Results of E-tests of multivariate normality per morphospecies and generation. Figure S1. Comparison of the morphospace occupation reconstructed by non-metric multidimensional scaling (nmMDS) in 2 and 3 dimensions along nmMDS1 and 2, with vectors representing how individual specimens are displaced from the 3D configuration to the 2D configuration. Figure S2. Geometric morphometric shape changes along nmMDS1 and 2. [file 12862_2019_1570_MOESM1_ESM.docx]

**Additional File 1: Supplementary information to the main text, consisting of supplementary text, table S1, figures S1 and S2.**

**Supplementary text.**

**Additional sampling information.** At each locality, a shoreline stretch of ~500 m was sampled from the shore up to 5 m depth, with sieves attached to a telescope stick, and by hand while wading and snorkeling, except in the Shire River where wading or snorkeling was prevented by the presence of crocodiles and hippopotami. We collected adults rather than juveniles for several reasons. First, the identification of juveniles to morphospecies is often ambiguous, complicating the design of the experiment. Second, rearing wild-caught juveniles to maturity in a common laboratory environment may affect the shell morphology of both parents and offspring, which would confound the experiments. Third, *Lanistes* populations in the Malawi Basin have a skewed age distribution with very few juveniles and abundant adults [1], limiting the feasibility of collecting adequate numbers of juveniles.

**Setting up aquaria.** Each aquarium was filled with about 4 cm of sterilized river sand, a few small stones and a piece of hardwood as substrate and topped up with tap water. Aquaria were planted with some submerged aquatic macrophytes at first, but these plants were rapidly eaten, and not replaced. The water was treated with pH-plus (JBL, Neuhofen, Germany), and water hardness was buffered with *Sepia* cuttlebone and bagged coral fragments. Furthermore, each tank had a 75 W heating element, a Biopower 160 filter and a single air diffuser with a flow of 100 L/hour (all from Eheim, Deizisau, Germany). To help maintain the ecological equilibrium of individual tanks, a few plecostomid fishes (*Ancistrus gastrosteus*) from a commercially-obtained inbred lineage were added to each tank.

Animals were fed daily (morning and evening) *ad libitum* with JBL Novo Pleco chips, additionally two to three times per week with a suspension of freeze-dried phytoplankton (SBAE industries, Evergem, Belgium), and occasionally with a supplement of vegetables (carrot, cucumber, lettuce). Pumps were cleaned as needed (at least weekly) to guarantee a continuous high level of mechanical and biological filtration, and 1/3 of the water was changed every 2-3 weeks to avoid the buildup of metabolites. Aquaria were all located in the same preheated laboratory space, with room temperatures usually ~20 °C, but varying seasonally between 17 and 27 °C. All aquaria were aligned on desks along the west-northwest facing window side of the lab, so that they experienced the diurnal cycle in Belgium, and each tank obtained a limited period of direct sunlight for the second half of the afternoon. To avoid any effect of individual aquaria or their position on the experiment randomizations were performed in two ways. First, snails (but not the fish) were transplanted every two months between aquaria and second, aquaria were translocated in position every four months.

**Extended procedures required by ampullariid biology.** The ampullarid *Lanistes* is dioecious with internal fertilization, and females have a seminal receptacle [2], implying that wild-caught females may store sperm from previous mating in the wild. If morphospecies mate assortative, this aspect would not complicate our study whereas non-assortative mating could have a homogenizing effect. Mate-choice preferences for *Lanistes* in the wild are unknown, and therefore, we took precautions to prevent that offspring in the lab resulted from fertilization with sperm from unknown males in the wild. First, females were provided with opportunities to produce fertile eggs in isolation for 10 days (i.e. during the acclimatization phase). No egg clusters were formed during this period, and we have never observed the production of an egg cluster without copulation in the preceding three days. This observation differs markedly from other ampullariids, e.g. *Pomacea*, whose females may produce fertile eggs in isolation up to several weeks after their last copulation [3]. The observations on our *Lanistes* species could have various causes: females may not store sperm that long, they may not have been able to maintain sperm during the stressful conditions after capture and before arrival in the lab (during which no copulations nor the deposition of egg clusters have been observed), or they may still store sperm but delay its use until new sperm transfers take place, i.e. upon copulation.

After the isolation phase, lab populations were formed in which individuals could mate freely and produce eggs, but to avoid an influence of pre-experimental mating we removed egg clusters formed in the first month of the experiment. This precaution is reasonable because sperm competition has been described in ampullariids, and mate-switching experiments have demonstrated a rapid turnover (days up to weeks) in the paternity of offspring towards the most recent partner [4]. Additional evidence for the limited capacity of sperm storage in our *Lanistes* species was obtained from hybridization experiments between *L. nyassanus* and *L.* sp. (*ovum*-like) (Van Bocxlaer, unpublished data). Males and females of these morphospecies were paired in a hybridization experiment with forced-mating design one month after the common garden experiment, and although viable *F_1_* offspring was produced by some of these interspecific pairs, several females produced unfertilized morphologically-aberrant eggs only. Such eggs were never observed during the common garden experiment, and if sperm storage for delayed fertilizations were important, these females should have produced at least initially some fertilized eggs in the hybridization experiment.

**Data collecting.** Shells were digitized in apertural view using a Nikon D3100 or Canon Eos 350D SLR camera with a Nikkor Micro 60 mm or a Canon EFS 60 mm macro lens, respectively. Specimens were oriented with the spiral axis horizontal and the aperture so that a tangent perpendicular to the horizontal plane could be constructed to the most distal point of the apertural margin [5]. Photos were rotated and cropped in Adobe Photoshop CS5 before they were compiled in a .tps file using TpsUtil v. 1.75 [6] for digitization in TpsDig v. 2.31 [7]. For a discussion on replication error, see Van Bocxlaer & Schultheiß [5].

**Model-based clustering.** Here we describe the spherical and diagonal models fit to our morphospace data. The underlying mathematical models make different assumptions of how the data would be distributed per group, and of how the volume and shape of the variance compares among groups.

**Model identifier Distribution Information**

EII Spherical Equal volume, equal shape

VII Spherical Variable volume, equal shape

EEI Diagonal Equal volume, equal shape

EVI Diagonal Equal volume, variable shape

VEI Diagonal Variable volume, equal shape

VVI Diagonal Variable volume, varying shape

**Results.**

**General observations on experiments.** Upon formation of experimental populations and shortly after copulation, females started depositing mucus-embedded egg clusters on the aquarium walls, heating element and filter. Egg cluster deposition occurred in bursts in our experiment, probably because young juveniles, which emerge from the egg after ~14 days, continue feeding as long as possible on the mucus and eggs available in the aquarium, therewith drastically reducing the survival of offspring from egg clusters that were deposited later.

**References**

1. Louda SM, McKaye KR: **Diurnal movements in populations of the prosobranch *Lanistes nyassanus* at Cape Maclear, Lake Malawi, Africa**. *Malacologia* 1982, **23**:13-21.

2. Berthold T: **Vergleichende Anatomie, Phylogenie und historische Biogeographie der Ampullariidae (Mollusca, Gastropoda)**. *Abh Naturwiss Ver Hamburg* 1991, **29**:1-256.

3. Albrecht EA, Carreño NB, Castro-Vazquez A: **A quantitative study of environmental factors influencing the seasonal onset of reproductive behaviour in the South American apple-snail *Pomacea canaliculata* (Gastropoda: Ampullariidae)**. *J Molluscan Stud* 1999, **65**:241-250.

4. Yusa Y: **Inheritance of colour polymorphism and the pattern of sperm competition in the apple snail *Pomacea canaliculata* (Gastropoda: Ampullariidae)**. *J Molluscan Stud* 2004, **70**:43-48.

5. Van Bocxlaer B, Schultheiß R: **Comparison of morphometric techniques for shapes with few homologous landmarks based on machine-learning approaches to biological discrimination**. *Paleobiology* 2010, **36**:497-515.

6. Rohlf FJ: **tpsUtil v. 1.75**. In*.*, 1.60 edn: Department of Ecology and Evolution and Anthropology, State University of New York at Stony Brook, NY; 2018.

7. Rohlf FJ: **tpsDig 2 v. 2.31**. In*.*, 2.16 edn: Department of Ecology and Evolution and Anthropology, State University of New York at Stony Brook, NY; 2017.

**Table S1.** Results of *E*-tests of multivariate normality per morphospecies and generation. The null hypothesis that the data derive from a multivariate normal distribution is overall not rejected, except for *L. nyassanus F_2_* (*p*-value indicated in bold). G = generation, with ‘O’ or ‘P’ indicating offspring or parents, respectively.

| **Morphospecies** | **G** | **energy** | ***p*-value** |
| --- | --- | --- | --- |
| *L. nyassanus* | *P* | 0.566 | 0.627 |
| *L. solidus* | *P* | 0.583 | 0.652 |
| *L.* sp. (*ovum*-like) | *P* | 0.858 | 0.113 |
| *L. nyassanus* | *F_1-O_* | 0.753 | 0.239 |
| *L. solidus* | *F_1-O_* | 0.662 | 0.433 |
| *L.* sp. (*ovum*-like) | *F_1-O_* | 0.561 | 0.716 |
| *L. nyassanus* | *F_1-P_* | 0.460 | 0.932 |
| *L. solidus* | *F_1-P_* | 0.584 | 0.650 |
| *L.* sp. (*ovum*-like) | *F_1-P_* | 0.661 | 0.392 |
| *L. nyassanus* | *F_2-O_* | 0.967 | **0.044** |
| *L. solidus* | *F_2-O_* | 0.678 | 0.351 |
| *L.* sp. (*ovum*-like) | *F_2-O_* | 0.579 | 0.631 |


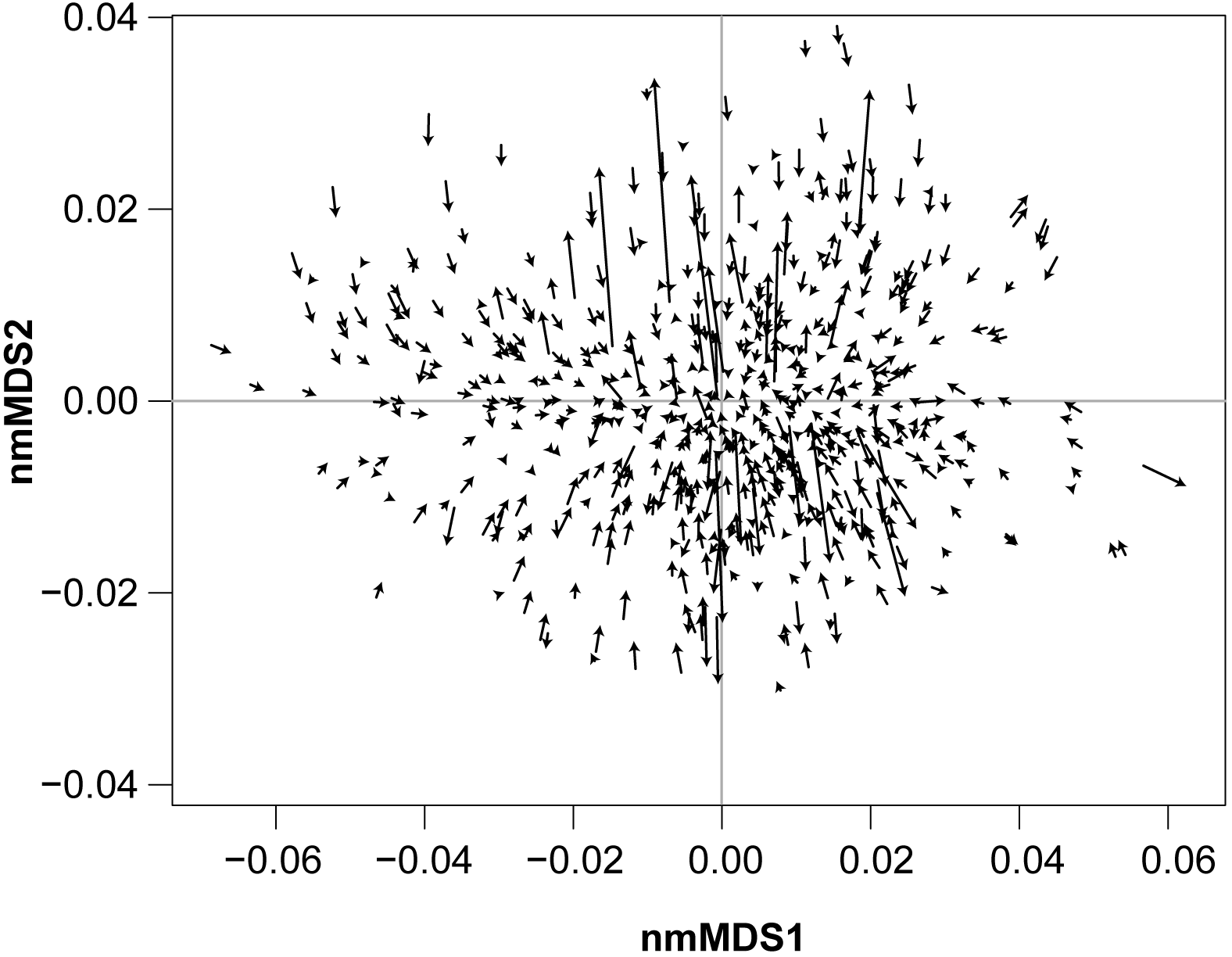


**Figure S1.** Comparison of the morphospace occupation reconstructed by non-metric multidimensional scaling (nmMDS) in 2 and 3 dimensions along nmMDS1 and 2, with vectors representing how individual specimens are displaced from the 3D configuration towards the 2D configuration. Overall the figure displays minimal changes as to the morphospace occupation, with only a few large displacements along nmMDS2. There are no major displacements from one quadrant of the morphospace to another, nor do the relatively large displacements concern a single experimental group of specimens. Therefore, these changes do not affect biological interpretations and nmMDS in 2D is robust for our dataset.


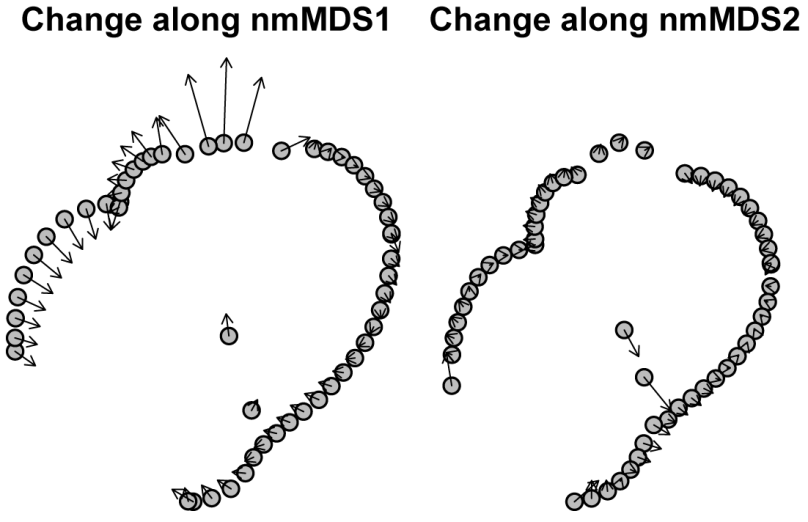


**Figure S2.** Shape change along nmMDS1 and 2. The configuration of circles indicates the shape at the negative extreme of the axis with arrows indicating the displacement towards the positive extreme while maintaining a position at the origin of the other axis, e.g. shape changes along nmMDS1 are taken at nmMDS2 = 0 and vice versa.
